# Written communication of whole genome sequencing results in the NHS Genomic Medicine Service: a multi-centre service evaluation

**DOI:** 10.1038/s41431-024-01636-5

**Published:** 2024-05-28

**Authors:** Holly Ellard, Angus Clarke, Sarah Wynn, Amanda Pichini, Celine Lewis

**Affiliations:** 1 School of Medicine, Cardiff University, Cardiff, Wales UK; 2 Population, Policy and Practice Department, UCL Great Ormond Street Institute of Child Health, London, UK; 3 Division of Cancer & Genetics, School of Medicine, Cardiff University, Cardiff, Wales UK; 4 Unique, Rare Chromosome Support Group, Oxted, Surrey UK; 5 Genomics England, London, England UK; 6 NHS North Thames Genomic Laboratory Hub, Great Ormond Street Hospital for Children NHS Foundation Trust, London, UK

**Keywords:** Genetic testing, Medical genomics

## Abstract

Whole genome sequencing (WGS) is being used in diagnostic testing for certain clinical indications within the NHS Genomic Medicine Service (GMS) in England. Letter writing is an integral part of delivering results. However, no national guidelines for writing results from WGS exist. This multi-centre service evaluation used mixed methods to understand the content and readability of letters returning diagnostic, variant of uncertain significance (VUS), and no-finding results to paediatric rare disease patients. Eight Regional Genetics Services (response rate 47%) in England provided a total of 37 letters returning diagnostic (*n* = 13), VUS (*n* = 10), and no-finding (*n* = 14) results. Diagnostic and VUS results were usually delivered during an appointment; no-finding results were typically delivered by letter only. Letters were diverse in which content topics they covered and level of detail. No-finding letters (14/14) explained the result but were less likely to cover other topics. Diagnostic letters discussed the result (13/13), the condition (13/13), clinical genetics follow-up (13/13), clinical management (10/13), and adapting to the result (9/13). VUS letters explained the result (10/10), diagnostic uncertainty (10/10), and clinical genetics follow-up (10/10). Uncertainty was a common component of letters (33/37), irrespective of the result. Reanalysis or review after one or more years was suggested in 6/13 diagnostic, 7/10 VUS, and 6/14 no-finding letters. The mean reading level of letters corresponded to 15–17 years. Understanding how WGS results are conveyed to families during appointments, as well as how families interpret that information, is needed to provide a more comprehensive overview of results communication and inform best practices.

## Introduction

In 2018, the Genomic Medicine Service (GMS) was introduced into the National Health Service (NHS) in England to implement whole genome sequencing (WGS) as a diagnostic test for certain rare diseases and cancers [1]. Most (50–75%) rare diseases affect children; these conditions are often multisystemic with genetic and phenotypic heterogeneity, making a diagnosis challenging to achieve [2]. In WGS, multiple genes are simultaneously analysed through virtual panels of known genes associated with a patient’s phenotype, which may contain hundreds of genes. This testing approach has shortened the diagnostic journey for many paediatric patients [3]. The types of results that a patient may receive from WGS through the GMS include: a diagnostic result, where a variant is found that explains the patient’s clinical presentation and can be used to make a diagnosis; a no-finding result, where no pathogenic or clinically relevant variants are found; or a variant of uncertain significance (VUS) result, where there is insufficient or contradicting evidence to discern the pathogenicity of a variant. A VUS cannot be used to make a diagnosis or inform clinical management but may be reclassified in the future with new information.

Letters to patients are an integral part of delivering results in clinical genetics [4]. Patients typically receive a summary letter of their result appointment. Alongside providing a written record of the outcome of testing, letters aid information recall, provide means to share information with family members, facilitate access to management options, promote feelings of autonomy, signpost reliable information, and improve the accuracy of risk perception [5–7]. To support the communication of results in the GMS, a cross-professional competency framework has been developed that outlines the professional knowledge, behaviours, and skills required to convey genomic results [8]. However, there are currently no national guidelines or templates for writing genomic results letters to patients and there are few published studies exploring patient preferences about summary letters in clinical genetics to draw conclusions about effective letter-writing practice [5, 7, 9].

Compared to previous genetic testing, the comprehensive nature of WGS has amplified existing challenges for healthcare professionals returning test results [10–12]. These include navigating diagnostic results with limited information about prognosis and a lack of patient support groups, conveying the nuanced meaning of no-finding results, and facilitating an understanding of VUS results and their implications [10–12]. The additional complexities of WGS, as well as the need to communicate complicated biological topics in a way that supports individuals with low health literacy [13], may pose a barrier to effective and accessible letter writing.

As the UK government is committed to sequencing 500,000 whole genomes by 2024 [14], it is imperative to define current practice of results communication in the NHS GMS to identify strengths, weaknesses, and opportunities for improvement. This mixed methods multi-centre service evaluation aimed to characterise and evaluate the written communication of results to patients in the GMS, with a focus on paediatric rare diseases. Specifically, we sought to investigate the topics covered in patient letters returning WGS results and to assess the readability of those letters.

## Methods

### Methodological approach

Our mixed methods study design utilised qualitative and quantitative methods to understand the content and readability of letters returning diagnostic, VUS, and no-finding results from WGS.

### Participants and recruitment

Participants, namely consultant geneticists and clinical genetics registrars who had returned results from WGS to paediatric rare disease patients, were invited from each of the 17 Regional Genetics Services (RGSs) located across England. At each RGS, one consultant geneticist and one clinical genetics registrar were asked to provide three de-identified letters (one for each result type) addressed to paediatric patients and their families that returned results from WGS for intellectual disability (code R29 in the National Genomic Test Directory v5.2 [15]). This clinical indication was specified a priori as it is an indication for which WGS is frequently ordered. Other indications were acceptable if the participant had not returned an R29 result. Incidental findings were not in scope for this study as they occur infrequently, so it was expected that participants were less likely to have letters returning these results. Participants were asked to complete a survey to characterise sample demographics and usual practice of returning WGS results. This survey was informed by variables investigated in empirical studies of written communication in genetics [16, 17] and input from the study team. Data collection occurred between January 2023 and April 2023.

### Data analysis

Letters were grouped according to the result they returned (diagnostic, VUS, or no-finding) and analysis was performed independently for each group (as described below). Findings from each strand were synthesised to address the overall study aim [18].

Inductive content analysis [19] was used to characterise the topics covered in letters. This involved two rounds of coding. In the first round, text was coded into broad topics. In the second round, coded text was interrogated in a line-by-line fashion to describe each topic in more detail. The coding process was non-linear and reflective, with codes being compared and amended between texts and as analysis proceeded. The number of letters containing a code within each letter group was counted to support interpretation. Three letters (one for each result type) were independently coded by HE and CL to compare results and resolve discrepancies. HE analysed the remaining letters.

This study used the Flesch–Kincaid formula to predict the reading grade level of letters [20], which calculates reading grade level as below:

$$0.39\left(\,\frac{{total\; words}\,}{{total\; sentences}}\right)+11.8\left(\frac{{total\; syllables}}{{total\; words}}\,\right)-15.59$$

The formula predicts reading grade level for 75% comprehension of the text. This formula was chosen for its widespread acceptability and use in existing relevant literature [16, 21]. To prevent overestimations, defined medical terms were replaced with the word ‘cat’ [16, 21], and full-stop punctuation marks that did not signify the end of a sentence were removed prior to applying the formula. Reading grade level output is based on U.S. school grades, therefore, the ages of students in U.S. grades were used to contextualise output for UK readers. Letters were also assessed for the inclusion of images and signposting to other sources of information.

## Results

Of the 17 RGSs contacted, one did not respond, one local governance team was unreachable, two were unable to identify clinicians willing to participate, two ceased contact during site-specific governance processes, and one was unable to complete site-specific governance processes in time, meaning that we received approval for the study from 10 RGSs. Two RGSs were unable to send data in time for analysis, yielding eight participating RGSs (47% response rate). A total of 37 de-identified letters were collected from 14 clinicians: 13 diagnostic result letters, 10 VUS result letters, and 14 no-finding result letters from WGS testing for intellectual disability (R29) (*n* = 20) or congenital malformation and dysmorphism syndromes (R27) (*n* = 7). For 10 letters, participants did not state the indication for testing. Eleven of the 14 participants completed the participant survey (Table 1). Most (6/11) participants spent 15 to 30 minutes writing letters and 27% (3/11) had access to departmental letter templates for a no-finding result. It was usual practice to return diagnostic (for 11/11 participants) and VUS (10/11) results during an appointment, whereas no-finding results were usually returned via letter only (9/11).

**Table 1:** Participant survey responses about participant characteristics and usual practices of writing whole genome sequencing result letters.

Characteristic		Frequency (*n* = 11)	Percentage
Profession	Consultant	10	91%
	Registrar	1	9%
Years in practice	2–5	1	9%
	6–10	2	18%
	>10	8	73%
Most commonly seen patients for WGS	Paediatric	11	100%
	Prenatal	2	18%
	General (adult)	2	18%
Involvement in 100,000 Genomes Project	Yes	10	91%
	No	1	9%
Departmental letter templates	Yes	3	27%
	No	8	73%
Type of template letter provided	No-finding result	3	27%
Time spent writing letters	<15 min	4	36%
	15–30 min	6	55%
	30–45 min	2	18%
	>60 min	1	9%
Usual mode of diagnostic result delivery	Telephone	1	9%
	Video	4	36%
	In-person	9	82%
Usual mode of no-finding result delivery	Telephone	2	18%
	Video	2	18%
	In-person	1	9%
	Letter only	9	82%
Usual mode of VUS result delivery	Telephone	1	9%
	Video	7	64%
	In-person	7	64%
	Letter only	1	9%

*WGS* whole genome sequencing, *VUS* variant of uncertain significance.

Ten content topics were identified:

1. Introduction and closing
2. Clinical summary of the patient
3. Explanation of test
4. Explanation of result
5. Biological information
6. Implications for the patient
7. Implications for family members
8. Clinical genetics follow-up
9. Adapting to the result
10. Uncertainty arising from the result

**Fig. 1:**
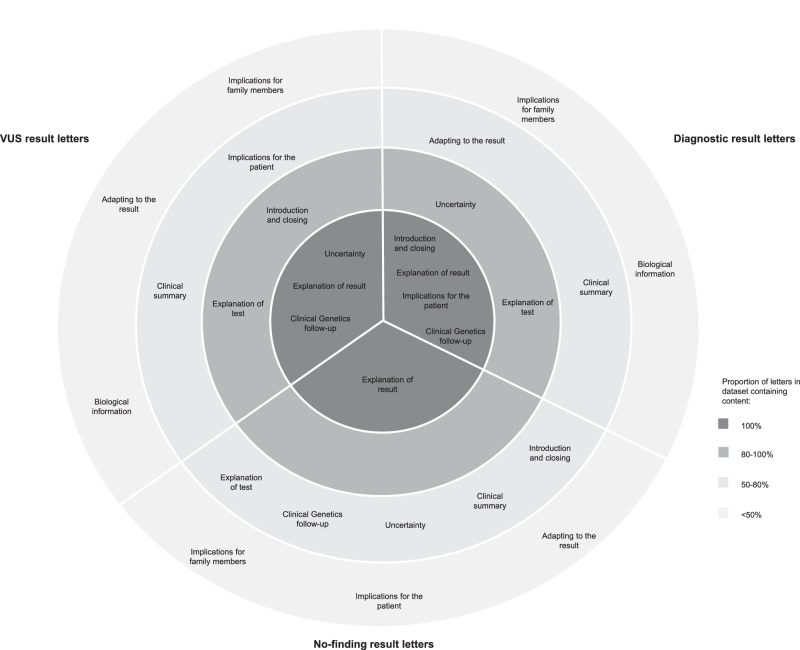
A circle map summarising the relative frequency at which content topics were included in patient letters returning whole genome sequencing results. The circle map is divided into letters returning diagnostic results (*n*=13), VUS results (*n*=10), and no-finding results (*n*=14). Within each division, topics in the inner most circle were included in 100% of letters returning that result type; topics in the second circle from the centre were included in 80-100% of letters; topics in the third circle from the centre were included in 50-80% of letters; and topics in the outer most circle were included in <50% of letters.

Letters were diverse in which topics they covered and the level of detail they went into. The proportion of letters that covered each topic is summarised in Fig. 1. Diagnostic and VUS letters contained similar topics, for example, over 80% of letters contained an explanation of the test, an explanation of the result, clinical genetics follow-up, and uncertainty arising from the result. There were fewer similarities between no-finding letters, with several topics covered in less than 50% of letters.

## Explanation of the result

In diagnostic letters (13/13), explanations included a statement of the genetic diagnosis, information about the altered gene, and whether the variant was de novo or inherited. Most variants were de novo (11/13) and explained in terms of absence in parental samples (Table 2: Q1). Most letters provided details about the variant (Table 2: Q2).

**Table 2:** Illustrative quotes from inductive content analysis of letters returning diagnostic, variant of uncertain significance, and no-finding results from whole genome sequencing.

	Result type	Content topic	Content subtopic	Illustrative quote from letter
Q1	Diagnostic	Explanation of the result		“…gene change was not detected in either of you indicating that it had occurred as a new or de novo change”
Q2	Diagnostic	Explanation of the result		“this change was predicted to have a significant effect on the gene”
Q3	VUS	Explanation of the result		“this specific change has not been described before but nor is it present in the large data sets available for the lab to compare with”
Q4	Diagnostic	Biological information		“We all have several genes that have a misprint in them: some of them are simply part of the benign variation of the general population… Only some misprints might cause specific congenital and/or medical problems”
Q5	Diagnostic	Implications for the patient	Explanation of the condition	“there is limited information about the condition but I have looked at all papers I could find and summarised the key points”
Q6	VUS	Implications for the patient	Explanation of the condition	“I was able to show you some information about the type of difficulties and medical issues that individuals who have definite X gene change suffer from… we are not aware that X has any of these… but, as he has never had a brain scan, I have asked Dr X if he would arrange this”
Q7	Diagnostic	Implications for the patient	Inheritance	“this means there would be a 1 in 2 (50%) chance of inheriting this condition”
Q8	Diagnostic	Implications for the patient	Inheritance	“the chances of X having a child with the same condition is very low”
Q9	No-finding	Implications for the patient	Inheritance	“we still feel that X has a recessive condition”
Q10	Diagnostic	Implications for the patient	Clinical management	“Children with this condition may also develop problems with their kidneys, hearts and eyes and hence, I suggested that it would be useful to refer X for these systems assessment.”
Q11	Diagnostic	Implications for family members	Recurrence risk	“…likely to be relatively small, probably in the region of around 0.5-1% (1 in 200 to 1 in 100)”
Q12	Diagnostic	Implications for family members	Recurrence risk	“I explained that there is a small risk that the X gene change could be present in one of you but not in the cells found in the blood”
Q13	VUS	Implications for family members	Recurrence risk	“the chance of having another child with this condition is 1 in 4”
Q14	No-finding	Implications for family members	Recurrence risk	“we cannot accurately advise on the chance of a further pregnancy being similarly affected”
Q15	No-finding	Implications for family members	Recurrence risk	“the chance of each of your future pregnancies having X is small”
Q16	Diagnostic	Clinical genetics follow-up		“I would like to see X again in three to five years’ time just in case there is more information found out about the condition”
Q17	Diagnostic	Clinical genetics follow-up		“you kindly gave us permission to write up his report for publication… so that other people can benefit from learning”
Q18	No-finding	Adapting to the result		“I appreciate that the lack of an underlying diagnosis may be frustrating, it may also in some ways be a relief”
Q19	Diagnostic	Adapting to the result		“there is nothing that you have done that has caused this and nothing that you could have done that would have prevented this”

In VUS letters (10/10), explanations included information about the altered gene and details about the variant (Table 2: Q3). In 3/10 VUS letters, it was explicitly stated that the outcome of WGS was that no genetic cause had been identified. These letters framed the VUS as ancillary to a primary no-finding outcome and affirmed that the VUS was unlikely to be causative.

Explanations of no-finding results involved a statement that WGS had not identified a genetic cause for the patient’s phenotype. Language was sometimes used that conveyed a clinician’s point of view toward the result; in 1/14 letters, the result was described positively as *'normal'* and *'good news'* that the clinician was *'pleased'* to give. In 6/14 letters, the result was described negatively as a *'negative'* outcome from testing (5/14), by explaining that the test had *'failed'* to identify a cause (1/14), and by introducing the result statement with *'unfortunately'* (1/14).

## Biological information

Contextual biological information was provided in 3/13 diagnostic letters and in 2/10 VUS letters, such as nuanced descriptions of cells, genes, chromosomes, and/or genetic variation (Table 2: Q4).

## Implications for the patient

### Explanation of the condition

Diagnostic letters (13/13) described the features associated with the genetic diagnosis. Several letters (8/13) recognised the novelty and hence limited knowledge surrounding the condition and, in turn, summarised relevant scientific papers (Table 2: Q5). Most diagnostic letters (11/13) enclosed or signposted an information resource about the condition. These were scientific articles (8/13) or information produced by charities (4/13). In 5/10 VUS letters, features associated with pathogenic variation in the altered gene were summarised, one of which signposted a scientific article for reference. A comparison between this information and the patient’s phenotype was often made and, in some cases, used to explain the purpose of follow-up phenotypic investigations (Table 2: Q6).

### Inheritance

In 6/13 diagnostic letters, the inheritance pattern of the condition was stated or explained in the letter or an attached leaflet. A quantitative or qualitative description of inheritance risk was given in 8/13 letters (Table 2: Q7, Q8). One VUS letter and one no-finding letter provided a suspected inheritance pattern (Table 2: Q9).

### Clinical management

In 10/13 diagnostic letters, 2/10 VUS letters, and 3/14 no-finding letters, clinical management was mentioned. In diagnostic letters, the result influenced management by facilitating onward referrals based on what is known about the condition (Table 2: Q10). Otherwise, patients continued to be managed according to clinical need. In VUS and no-finding letters, the result did not influence management, which continued to address patients’ symptoms.

## Implications for family members

### Recurrence risk

In 6/13 diagnostic letters, a qualitative and/or quantitative description of recurrence risk was provided to parents (Table 2: Q11). Residual risk was attributed to unexcluded mosaicism in 5/13 letters (Table 2: Q12). Letters that did not provide recurrence risk often recalled that parents were not planning further children. One VUS letter provided a quantitative description of recurrence risk based on suspected inheritance (Table 2: Q13). In 3/14 no-finding letters, it was explained that recurrence risk was low or unknown (Table 2: Q14, Q15).

### Reproductive options

In 4/13 diagnostic letters, the reproductive options available to parents were stated or explained. A leaflet on genetic testing options in pregnancy was enclosed in 2/13 letters.

### Siblings

In 4/13 diagnostic letters, the implications of the result for siblings were discussed, which reassured parents that siblings were unlikely to have the condition and offered genetic testing to exclude this in 2/13 letters.

## Clinical genetics follow-up

**Fig. 2:**
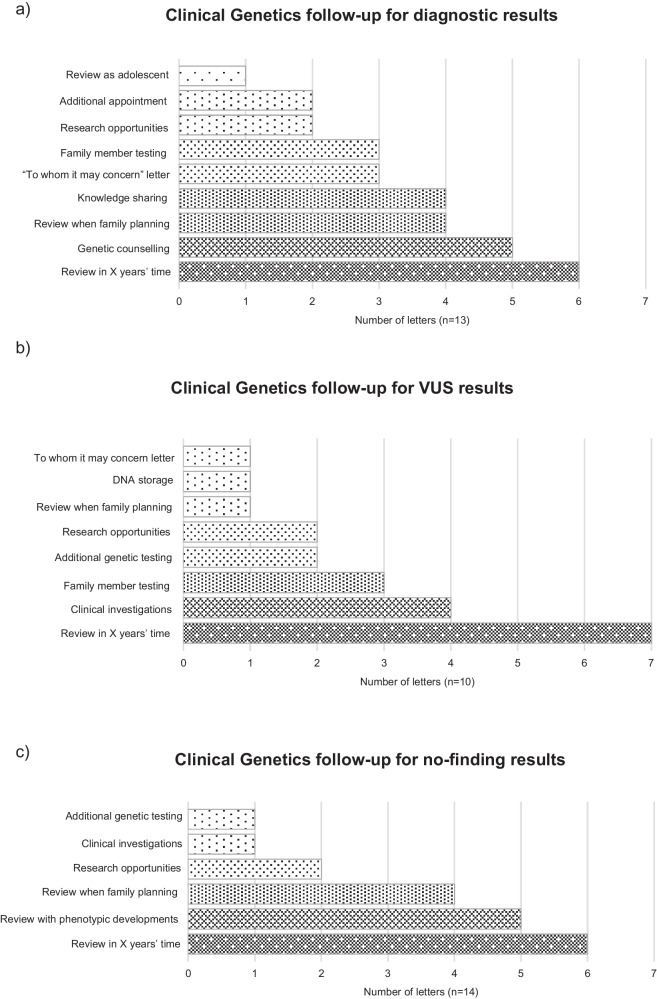
The type and frequency of clinical genetics follow-up described in letters returning whole genome sequencing results. Follow-up reported in (**a**) diagnostic result letters, (**b**) variants of uncertain significance result letters, and (**c**) no-finding result letters.

Follow-up plans with clinical genetics after results disclosure were discussed in 13/13 diagnostic letters, 10/10 VUS letters, and 11/14 no-finding letters. Across letters, the most frequent follow-up option was patient review after a certain number of years had passed (Fig. 2). In diagnostic letters, this was to allow for the accumulation of knowledge relating to the condition and ranged from two to five years (Table 2: Q16). In VUS letters, this was to allow for improvements in knowledge and technology and ranged from one to four years. Other VUS letters actively sought evidence for variant reanalysis, such as further clinical data. In no-finding letters, this was to consider whether new genetic tests were available, if new panels could be applied to the data, and to allow for improvements in knowledge and ranged from one to five years. Genetic counselling was offered to several families with a diagnostic result to discuss reproductive options and answer questions, or for patients to learn about their condition in adulthood. Requests to share learning from the result via case report publication or discussion at clinical meetings were made in 4/13 diagnostic letters (Table 2: Q17).

## Adapting to the result

In 9/13 diagnostic letters, 1/10 VUS letters, and 3/14 no-finding letters, support was provided for the psychosocial issues arising from the result. In 3/13 diagnostic letters and one VUS letter, a specific issue raised by parents was described. These were concerns about a particular aspect of their child’s symptoms, such as challenging behaviour. In 6/13 diagnostic letters, a support group—either specific to the condition or a general rare disease organisation—or social media group related to the genetic diagnosis was signposted. In 4/13 diagnostic letters, additional support required in school settings was discussed. In 1/10 VUS letters and 3/14 no-finding letters, letters anticipated parents’ potential emotional responses to the result. For both result types, frustration and relief were anticipated (Table 2: Q18), and in no-finding letters, potential disappointment was expressed. One diagnostic letter anticipated feelings of guilt toward the genetic nature of the condition (Table 2: Q19).

## Uncertainty

Uncertainty was discussed in 12/13 diagnostic letters, 10/10 VUS letters, and 11/14 no-finding letters (Table 3). Prognostic uncertainty (the inability to supply a clear prognosis from the diagnosis) was discussed in diagnostic letters. Diagnostic uncertainty (the inability to yield a clear and accurate explanation for the patient’s clinical presentation) was discussed in diagnostic, VUS, and no-finding letters. Uncertainty was commonly managed through endeavours to reduce uncertainty.

**Table 3:** Uncertainty arising from diagnostic, variant of uncertain significance, and no-finding results from whole genome sequencing described in patient letters.

Result type	Dimension of uncertainty	Description	Number of letters	Illustrative quote(s) from letter
Diagnostic	Issue	Prognostic uncertainty (inability to supply a clear and accurate prognosis from the diagnosis)	12/13	“it could not be predicted how mildly or severely children might be affected”
	Source	Limited knowledge and data about the condition	7/13	“there is little data available about the outcomes in adults or life expectancy”
	Management	Emphasising diagnostic uncertainty	10/13	“The change in the gene causes it to malfunction with certainty and therefore, this is a secure diagnosis”
		Hope for future certainty	5/13	“…with emerging literature, I suspect that most of these previously described conditions are also likely to have a milder presentation which will become obvious in due course”
Diagnostic	Issue	Diagnostic uncertainty (inability to determine whether a specific feature could be attributed to the diagnosed condition)	3/13	“there are no known heart problems previously reported with this condition”
	Source	Limited knowledge about the condition or an alternative cause	2/13	“it is possible that his potential bicuspid aortic valve may be an extra detail that is not known about the condition” “Syndactyly of the 4^th^ and 5^th^ fingers is not reported in X disorder and may have a separate genetic or non-genetic basis or may be a very rare feature”
VUS	Issue	Diagnostic uncertainty (an inability to determine if the variant is causing the patient’s phenotype)	10/10	“This may simply be a healthy variant (of which we all have thousands) and not the cause… However, there is a small chance that it is the cause”
	Source	Insufficient evidence for accurate variant interpretation	10/10	“at present, there is not enough scientific evidence to confirm whether the change in the gene is harmful and, therefore, causative of X’s problems”
	Management	Hope for future certainty	9/10	“knowledge about genes and their variations constantly increases, and as new information becomes available we will hopefully be able to arrive at conclusive genetic diagnosis”
		Providing a clinical judgement about the likelihood of VUS causality	4/10	“this is likely to be harmless background change” “…identification of a further individual with the same gene change would be sufficient for it to be upgraded to pathogenic”
No-finding	Issue	Diagnostic uncertainty (an inability to confirm that the phenotype is not genetic in cause)	11/14	“…does not completely exclude the possibility of an underlying genetic condition” “this does not rule out a genetic problem”
	Source	Imperfect testing	7/14	“it is possible that he has a genetic condition that we are unable to detect with current technologies”
		Limited knowledge about genetic changes that cause disease	7/14	“it is still possible… caused by a change in a gene or genetic region not yet known to be associated with X”
		Possible multifactorial cause	3/14	“we need to consider that X’s difficulties do not have a single gene background. In other words, X’s difficulties may be multifactorial”
		Possible non-genetic cause	3/14	“it may be that X does not have a clear strong genetic factor causing his problems”
		Insufficient clinical data	1/14	“To help us investigate things further we would like to undertake a skeletal survey to see if there are any further clues”
	Management	Hope for future certainty	8/14	“as genetic technology improves, it is possible that future genetic testing could reveal a cause”

## Readability

The mean reading grade level for diagnostic, VUS, and no-finding letters was 11.06 [SD 1.8; range 9.0–14.4], 10.93 [SD 3.2; range 7.1–17.8], and 11.69 [SD 2.4; range 8.6–15.9], respectively. These means correspond to 16–17 years for diagnostic and no-finding letters and 15–16 years for VUS letters. No letters contained images.

## Discussion

To our knowledge, this is the first mixed methods study conducted to characterise written results communication in the NHS GMS for any testing indication. We found that content topics were not consistently covered in every letter, meaning that families received different types and amounts of written information about their results. Diagnostic and VUS letters were more likely to cover an explanation of the test, an explanation of the result, clinical genetics follow-up, and uncertainty arising from the result, with diagnostic letters further providing content on the implications of the result for patients. No-finding result letters tended to explain the result but were less likely to cover other topics.

Not all topics of information will be applicable to all clinical situations and families, which could explain why some topics were infrequently covered in letters in this study. However, it is notable and potentially concerning that less than a half of diagnostic letters provided parents with recurrence risk. Whilst we do not know if recurrence risk was discussed with families during appointments but not included in letters, it could be argued that this risk should be documented for parents in letters as part of their permanent record of information. Some diagnostic letters that excluded content on recurrence risk noted that parents were not planning further children, suggesting that information provision may have been tailored to individual families. This is supported by interviews with healthcare professionals about their experiences of disclosing genomic results, which found that triaging information relevant to patients was considered an important skill [10]. Even if it is not relevant to the family at the time, some parents may benefit from a record of recurrence risk in letters to account for changes in what information they perceive as important over time. For others, including this information may be inappropriate on account of what parents have disclosed during discussion in clinic. Understanding parents’ unique context is an important part of information provision that is sensitive and responsive to their individual needs.

The magnitude of genetic variation uncovered by WGS increases the risk of identifying variants that do not have a clear link with disease or have been observed in few or no other individuals, which has raised concerns about how to communicate uncertain information to families [22–24]. This study revealed that uncertainty was a common component of results communication and occurred irrespective of result type. According to Han’s conceptual taxonomy of uncertainties in clinical genome sequencing [25], clinicians in this study shared scientific uncertainties with families. These included issues of diagnostic uncertainty and prognostic uncertainty, along with the sources of uncertainty which were attributed to ambiguity or complexity (such as where conditional probabilities or multiple risk factors or outcomes diminish certainty) [25]. An ethical argument regarding the handling of uncertain genomic results stipulates that both the sources and issues of uncertainty should be communicated to enable families to engage in a process of uncertainty appraisal and adaptation [22], which supports the way that clinicians conveyed uncertainty in this study. This study indicated that emphasising what is certain as well as opportunities for reanalysis and review in the future as technology and knowledge of variants and conditions improve may help reduce potential negative impacts from the receipt of uncertain information. However, evidence-based recommendations on how to discuss and support the management of uncertainty for best patient outcomes are lacking. A scoping review of recommendations on how to communicate uncertainty during clinical encounters concluded that there is no ‘one-size-fits-all’ approach and strategies should depend on the goals of communication, the issues of uncertainty, and clinical scenarios to which the uncertainty pertains [26]. Given that uncertainty is a commonly discussed topic within genomic medicine, future research could explore the impact of uncertainty communication and management strategies on patient outcomes, which would benefit from using Han’s taxonomy [25] to consistently classify types of genomic uncertainty. This could inform guidance on how to tailor communication and coping strategies to individual patients and situations, although each case will require judgement on the part of the health professional.

Parents’ specific emotional responses to their child’s result as experienced in the consultation were not mentioned by letters in this study. In an interview study with 13 parents about their perceptions of genetic counselling summary letters [7], letters were considered an emotional support piece as well as an information resource, which suggests there may be value in acknowledging emotional responses to results and reaffirming coping strategies in letters. Connecting with families whose children have the same diagnosis has been found to be an important coping mechanism and learning opportunity in several studies [27–30]. Whilst a proportion of diagnostic letters signposted parents to support groups, no VUS or no-finding letters signposted a support group, despite the availability of support networks such as SWAN UK (run by Genetic Alliance UK) for families with an undiagnosed genetic condition [31]. This could help families receiving these results to adapt. Research is needed to understand the nature of psychological support that would be valuable to parents and how this may differ between those receiving a diagnostic, VUS, or no-finding result.

Letters sent to patients are only useful if they can be understood. In this study, a readability formula predicted that diagnostic and no-finding letters could be understood by those at a 16–17-year-old reading level, and VUS letters by those at a 15-16-year-old level. The target reading level for accessible health information is 9–11-years-old [32]; whilst we do not know the health literacy of the particular recipients of these letters, or if the letters were tailored accordingly, it could be argued that readability was poor. Poor readability has been reported among other studies that have assessed genetic counselling letters using the Flesch–Kincaid formula [16, 21], implying a wider issue within clinical genetics. Providing definitions for genetic terms in letters has been associated with improved readability [21]. Letters in this study often used undefined medical and genetic terms suggesting that providing definitions, such as in a glossary, could be a practical way to improve readability. Another strategy could be the use of images [16], which were not included in letters in this study. These could help patients to comprehend complex biological concepts, such as mosaicism. Several letters signposted or enclosed information leaflets or academic or charity publications. Whilst these were not analysed, academic publications are not written with lay audiences in mind and may not support understanding. Although, we do not know if prior information was provided to families to improve genomic literacy such as at the time of consent.

### Implications for letter-writing practice

This study found that diagnostic and VUS results are typically communicated to families during an appointment, meaning that summary letters form only one part of the information-giving process. In contrast, 82% of participants reported that no-finding results are usually returned by letter only, making letters the main source of information. Not all no-finding letters provided a clear follow-up plan or invited families to contact the service if they had concerns. This raises questions about how families are being supported to adapt to their results. Template letters for no-finding results could benefit practice by prompting the inclusion of these elements. Additionally, findings from this study indicate that template letters for diagnostic results could benefit from a prompt to include recurrence risk, if appropriate. Whilst it is important to tailor letters so that they are written with the particular recipient(s) in mind, common concepts arising in letters could also be templated, such as why some results are VUS and the potential for reanalysis. This could reduce the amount of time spent writing letters. Alongside traditional patient letters, alternative methods of results communication from genomic testing have emerged in the literature. These include patient-friendly genomic test report templates [33–35] and an e-booklet [36]. Summary letters could evolve to build on these resources with individualised information [37]. Co-design of future template resources involving relevant patient groups could help to ensure that the result reporting format, content topics covered, and terminology used are meaningful and understandable to families [34].

### Strengths and limitations

A strength of this study was its mixed methods design. However, data was not collected on whether letters were supplementary to, or substituted for, the oral delivery of results, nor was the information provided during consent appointments assessed, which may have impacted the content of letters. Furthermore, non-genetics clinicians were not included in this study but may be ordering WGS as part of mainstreaming genomic services within the NHS. To generate a fuller understanding of written communication, a future study might compare the verbal content of results delivery against summary letters, as well as how these relate to the information provided during consent appointments. Exploration of patients’ responses to results communication will be important to draw conclusions about effective practice.

## Conclusions

WGS has only recently been implemented as a clinical test for certain rare diseases in the NHS in England, and results from this test are only now beginning to be returned to patients. This multi-centre mixed methods study provided timely insight into written results communication in the GMS. Letters were diverse in the content they covered and were written at a higher-than-recommended reading level to support understanding. Our findings revealed how uncertainty is prevalent within genomic test results, including those where a diagnosis has been identified. Understanding how WGS results are conveyed verbally to families during appointments, as well as how families interpret that information, is needed to provide a more comprehensive overview of WGS results communication and inform best practices.

## Data Availability

This study used third-party data made available under agreements with participating NHS organisations restricting data availability. Reasonable requests for data access should be directed to the corresponding author (HE) to seek permission from participating NHS organisations.
